# 小鼠原代巨核细胞骨髓腔移植模型的构建

**DOI:** 10.3760/cma.j.issn.0253-2727.2022.04.002

**Published:** 2022-04

**Authors:** 柏铭 黄, 肖源 陈, 美娟 夏, 琳 郑, 翠翠 刘, 晶晶 赵, 培 苏, 洪涛 王, 家喜 周

**Affiliations:** 中国医学科学院北京协和医学院血液病医院（中国医学科学院血液学研究所），实验血液学国家重点实验室，国家血液系统疾病临床医学研究中心，细胞生态海河实验室，天津 300020 State Key Laboratory of Experimental Hematology, National Clinical Research Center for Blood Diseases, Haihe Laboratory of Cell Ecosystem, Institute of Hematology & Blood Diseases Hospital, Chinese Academy of Medical Sciences & Peking Union Medical College, Tianjin 300020, China

**Keywords:** 巨核细胞, 血小板, 骨髓腔移植, Megakaryocyte, Platelet, Intramedullary injection

## Abstract

**目的:**

利用小鼠原代巨核细胞骨髓腔移植构建血小板产生模型，为研究巨核细胞功能及血小板产生调控机制提供工具。

**方法:**

通过磁珠富集绿色荧光蛋白（Green Fluorescent Proteins, GFP）转基因供体小鼠骨髓原代巨核细胞，将其移植入经半致死剂量辐照的受体小鼠骨髓腔，建立原代巨核细胞骨髓腔内血小板生成模型。通过免疫荧光染色、流式细胞术等方法检测受体小鼠中供体来源巨核细胞和血小板形态、大小等指标。

**结果:**

磁珠富集可将巨核细胞在骨髓细胞悬液中的比例提高40～50倍。供体来源巨核细胞能够在受体小鼠骨髓腔成功定植并正常产生血小板，其产生的子代血小板具有与受体自身来源血小板相似的形态、大小及CD41、CD42d、CD61等表面标志分子表达水平。

**结论:**

通过磁珠富集与骨髓腔注射可成功构建小鼠原代巨核细胞移植模型，该模型能够客观反映巨核细胞在骨髓内产生血小板的能力。

## Abstract (en)

**Objective:**

To establish an intramedullary transplantation model of primary megakaryocytes to evaluate the platelet-producing capacity of megakaryocytes and explore the underlying regulatory mechanisms.

**Methods:**

Donor megakaryocytes from GFP-transgenic mice bone marrow were enriched by magnetic beads. The platelet-producing model was established by intramedullary injection to recipient mice that underwent half-lethal dose irradiation 1 week in advance. Donor-derived megakaryocytes and platelets were detected by immunofluorescence staining and flow cytometry.

**Results:**

The proportion of megakaryocytes in the enriched sample for transplantation was 40 to 50 times higher than that in conventional bone marrow. After intramedullary transplantation, donor-derived megakaryocytes successfully implanted in the medullary cavity of the recipient and produce platelets, which showed similar expression of surface markers and morphology to recipient-derived platelets.

**Conclusion:**

We successfully established an in vivo platelet-producing model of primary megakaryocytes using magnetic-bead enrichment and intramedullary injection, which objectively reflects the platelet-producing capacity of megakaryocytes in the bone marrow.

巨核细胞是血液系统中以产生血小板为主要功能的成熟细胞[1]。成年期巨核细胞主要存在于骨髓，约占骨髓有核细胞总数的0.05％～0.1％[2]。与其他血细胞相比，巨核细胞具有数量少、胞体大、胞质丰富等特点。较大的体积及胞质中丰富的内膜系统是成熟巨核细胞释放大量血小板的基础[3]–[5]，但同时也导致巨核细胞极易破碎，难以从体内被大量收集。因此，领域内对于巨核细胞的功能评估以及子代血小板产生机制的探索多采用体外诱导分化等手段进行研究[6]–[8]，针对体内原代巨核细胞进行探索的模型与工具应用有限。而随着近年来对于巨核细胞异质性解析的逐渐深入[9]，其功能评估的需求也随之增加，产生血小板作为巨核细胞最主要的功能，亟待稳定可靠的评估模型予以支持。本研究通过磁珠筛选对小鼠骨髓原代巨核细胞进行温和有效的富集后，以骨髓腔注射的方式进行巨核细胞移植，构建小鼠原代巨核细胞骨髓腔移植模型，拟用于评估巨核细胞体内产生血小板的能力，为探索巨核细胞功能以及子代血小板产生机制提供有力工具。

## 材料与方法

1. 主要试剂：兽用血细胞分析用稀释液购自深圳Mindray公司；台氏液购自广州沛瑜生物制品有限公司；磷酸盐缓冲液（PBS）、红细胞裂解液、0.5 mol/L乙二胺四乙酸（EDTA）溶液、4％组织细胞固定液、牛血清蛋白V（BSA）、DAPI荧光染料、Hoechst荧光染料购于北京索莱宝科技有限公司；杜氏磷酸缓冲液（DPBS）购于美国Gibco公司；TritonX-100购于上海碧云天生物技术有限公司；Biotin抗鼠CD41抗体、Streptavidin MicroBeads磁珠、LS磁珠分选柱购于德国美天旎生物科技公司；CD41、CD42d、CD61抗体均购自美国Biolegend公司。

2. 主要仪器：Canto Ⅱ流式细胞仪（美国BD公司产品），BC-5000Vet血细胞分析仪（深圳Mindray公司产品），转盘激光共聚焦显微镜（英国安道尔科技公司产品），CM1950冷冻切片机（德国徕卡公司产品）。

3. 实验动物：供体小鼠为8周龄绿色荧光蛋白（Green Fluorescent Proteins, GFP）转基因雄鼠，为C57BL/6j品系，其巨核细胞及其子代血小板均表达GFP荧光报告蛋白。受体小鼠为8周龄C57BL/6j野生型雄鼠。所有实验用鼠均为SPF级，由中国医学科学院血液病医院（血液学研究所）动物中心提供。

4. 小鼠巨核细胞富集：剥取小鼠股骨、胫骨、髂骨后，以1 ml注射器吸取DPBE缓冲液（含有2％胎牛血清与0.5 mol/L EDTA）冲取骨髓细胞，加入5 ml红细胞裂解液，4 °C静置5 min裂解红细胞，洗涤后以500 µl DPBE重悬，加入Biotin抗鼠CD41抗体4 °C避光孵育30 min，洗涤后加入Streptavidin MicroBeads磁珠再次4 °C避光孵育30 min。洗涤后以500 µl DPBE重悬。以1 ml PBS润洗LS磁珠分选柱后，将细胞悬液每次1 ml加入LS磁珠分选柱，加入全部细胞悬液后以1 ml DPBE冲洗，重复3次。收集吸附在LS磁珠分选柱上的CD41^+^细胞，取100 µl进行PE-CD41、APC-CD42d抗体孵育后，以流式细胞术检测CD41^+^CD42d^+^细胞比例。

5. 小鼠骨髓腔移植：提前1周以半致死剂量（4.5 Gy）对受体小鼠进行辐照处理。完成供体巨核细胞富集后，以20 µl PBS缓冲液重悬5×10^6^富集所得细胞。以10％水合氯醛麻醉受体小鼠，对其后肢进行剃毛备皮。以1 ml注射器针头于胫骨平台打孔定位，随后沿打孔处以1 ml胰岛素注射器将上述20 µl供体细胞悬液注射入受体小鼠胫骨骨髓腔。

6. 骨髓巨核细胞切片和原位免疫荧光染色：取受体小鼠注射侧股骨和胫骨浸没于4％组织细胞固定液过夜固定。洗涤后将骨组织浸于0.5 mol/L EDTA进行脱钙处理，24 h后浸于CPT溶液（含有30％蔗糖的1×PBS缓冲液）进行脱水处理。随后将完成脱水脱钙的骨组织包埋于EBM（含有8％明胶的CPT溶液），置于−80 °C过夜冷冻。最后用冰冻切片机以30 µm厚度缓慢切片并转移至组织防脱载玻片，待EBM融化蒸发后进行免疫荧光染色。

以疏水笔画框圈住骨髓组织所在位置，待完全晾干后于框内滴加PBS缓冲液将组织水化，5 min后吸除PBS，加入200 µl 0.3％ TritonX-100，室温静置20 min后吸除。加入1％ BSA溶液室温静置30 min进行封闭。将APC-CD41抗体按1∶100比例以1％ BSA稀释后滴加在组织表面，放入含有湿棉球的暗盒中，4 °C过夜进行孵育。次日以PBS洗涤3次，每次5 min。吸除PBS后滴加聚乙烯醇封片剂，盖上盖玻片，上机使用转盘激光共聚焦显微镜观察并记录骨髓中细胞形态。

7. 小鼠外周血血小板数量检测：自移植后48 h起至144 h，每24 h通过内眦静脉丛取血的方式取小鼠外周血10 µl，加入240 µl兽用血细胞稀释液后，轻弹混匀，上机使用血细胞分析仪以预稀释模式对血小板进行计数。

8. 小鼠血小板原位免疫荧光染色：在移植后96 h，以10％水合氯醛麻醉受体小鼠后，通过下腔静脉取血的方式收集外周血约800 µl。加入等体积常温生理盐水轻轻颠倒混匀，以198×*g*离心5 min，升降速均设置为5）。缓慢将上清转移至另一1.5 ml EP管，以1 200×*g*离心2 min；缓慢吸走上清后加入1 ml预热至37 °C的CGS缓冲液（含有0.72％氯化钠，0.66％葡萄糖与0.43％柠檬酸钠的去离子水溶液）重悬血小板。600×*g*再次离心2 min后重复CGS洗涤步骤，以200 µl常温台氏液重悬血小板沉淀。随后加入APC-CD41抗体和Hoechst荧光染料，室温避光孵育40 min后加入1 ml常温台氏液洗涤抗体，以1 200×*g*离心10 min后弃上清，以200 µl常温台氏液重悬沉淀，最终取20 µl血小板悬液加入共聚焦皿中央，静置沉降20 min后通过转盘激光共聚焦显微镜观察并记录血小板形态。

9. 流式细胞术检测小鼠外周血GFP^+^血小板：自移植后48 h起，每24 h通过内眦静脉丛取血的方式取小鼠外周血3 µl加入150 µl常温台氏液中，上机使用Canto Ⅱ流式细胞仪检测CD41^+^CD42^+^血小板群体中GFP^+^血小板群体的比例。

10. 流式细胞术检测血小板表面标志分子：在移植后96 h，以内眦静脉丛取血的方式采集受体小鼠外周血，取其中3 µl加入150 µl常温台氏液，随后加入APC-Cy7-CD41、PerCP-Cy5.5-CD42d及APC-CD61抗体室温避光孵育30 min后加入200 µl常温台氏液进行稀释，上机使用Canto Ⅱ流式细胞仪进行检测。

11. 统计学处理：使用FlowJo V10.4 软件对流式数据进行分析。使用GraphPad Prism8对各项实验数据进行统计作图。利用独立样本*t*检验对实验数据进行分析，其中*P*值小于0.05认为数据差异具有统计学意义。

## 结果

1. 供体小鼠骨髓原代巨核细胞富集效率及移植效果评估：在正常小鼠骨髓中CD41^+^CD42d^+^巨核细胞仅为0.05％～0.1％[2]。为获得纯度较高可供移植的骨髓原代巨核细胞，减少骨髓腔注射所需总液体量，本研究以GFP转基因小鼠作为供体，利用磁珠筛选对骨髓CD41^+^细胞进行富集（图1），最终所得细胞悬液中CD41^+^CD42d^+^巨核细胞比例达4％～5％，较富集前提高了近50倍（图2A、B、C）；且在剩余细胞中，CD41^+^CD42d^+^巨核细胞比例为0（图2D）。以上结果提示，通过该方式可有效捕获并富集骨髓巨核细胞。随后，我们将富集所得细胞注射入受体小鼠骨髓腔（图1）。为了研究供体巨核细胞的植入情况，我们在移植后48 h进行骨髓原位免疫荧光染色。观察发现，受体骨髓腔内可见供体来源GFP^+^CD41^+^巨核细胞，其具有正常巨核细胞的大小及形态（图3），表明磁珠富集与骨髓腔注射的方式足够温和，不会对巨核细胞造成过度刺激与伤害，能够保证其维持正常形态并成功定植于受体小鼠骨髓腔。

**图1:**
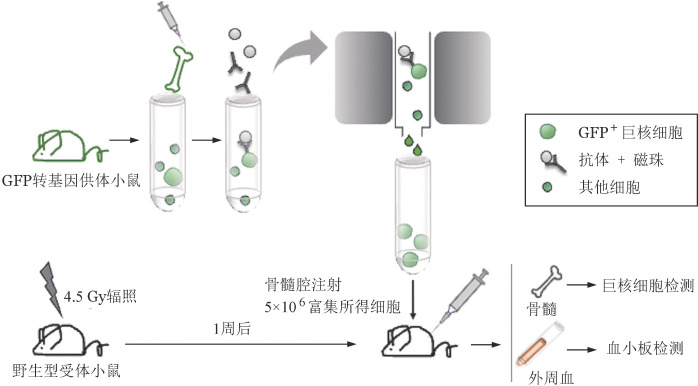
骨髓原代巨核细胞磁珠富集与小鼠骨髓腔移植模式图 GFP：绿色荧光蛋白

**图2:**
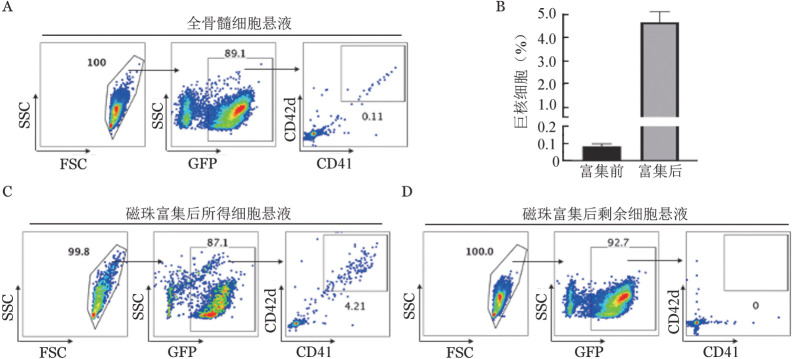
骨髓巨核细胞富集前后比例 A：流式细胞术检测富集前供体小鼠全骨髓细胞悬液中巨核细胞（CD41^+^CD42d^+^）比例；B：富集前后细胞悬液中巨核细胞比例对比（*n*＝3）；C：流式细胞术检测磁珠富集所得细胞悬液中巨核细胞比例；D：流式细胞术检测磁珠富集后剩余细胞悬液中巨核细胞比例。SSC：侧向角散射；FSC：前向角散射；GFP：绿色荧光蛋白

**图3:**
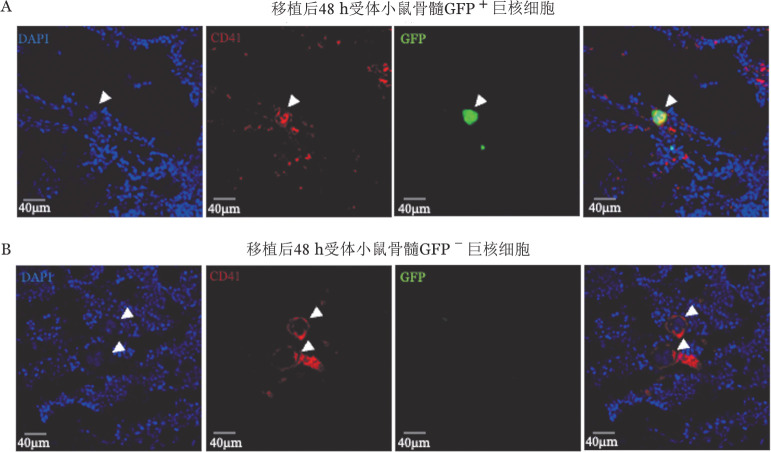
受体小鼠骨髓腔内巨核细胞形态 A：移植后48 h受体小鼠骨髓切片原位免疫荧光染色（白色箭头所示为供体来源GFP^+^巨核细胞）；B：移植后48 h受体小鼠骨髓切片原位免疫荧光染色（白色箭头所示为受体小鼠自身巨核细胞）。DAPI标记细胞核，GFP为供体小鼠细胞自发荧光

2. 供体巨核细胞在受体小鼠体内血小板产生情况：移植后72 h，受体小鼠外周血中开始出现少量GFP^+^血小板，移植后96 h其比例明显上升，在CD41^+^CD42d^+^血小板群体中出现明显的GFP^+^群体（图4A），该部分供体来源血小板总量可达到10×10^9^/L（图4B）。至移植后120 h，GFP^+^血小板在受体小鼠外周血中的比例达到峰值（图4A、C），此时受体小鼠外周血中血小板总量可达20×10^9^/L（图4B）。随后，随着受体小鼠自身造血功能的恢复和供体巨核细胞的消耗，供体来源的GFP^+^血小板所占比例开始下降。上述结果表明，原代巨核细胞定植于受体小鼠骨髓腔后，可成功在受体小鼠体内发挥其血小板产生功能。

**图4:**
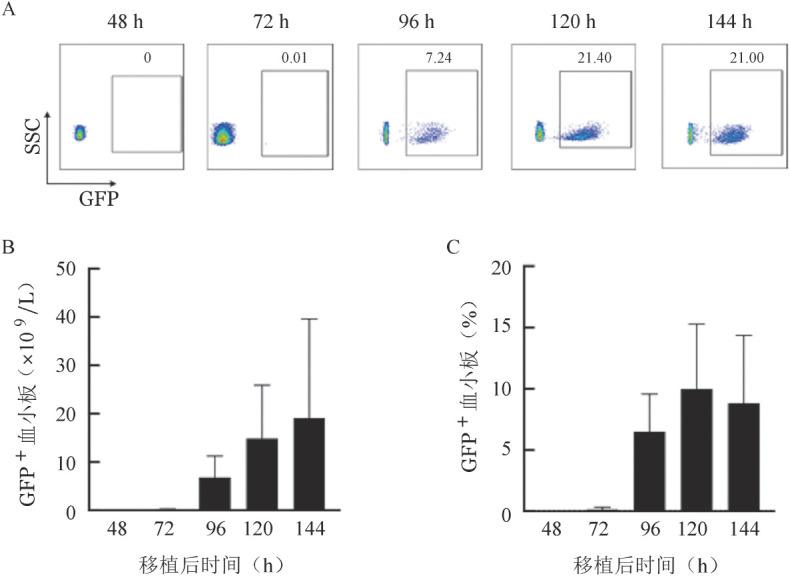
移植后受体小鼠外周血中供体来源血小板动态变化 GFP：绿色荧光蛋白；SSC：侧向角散射。A：流式细胞术检测移植后受体小鼠外周血中供体来源血小板（CD41^+^CD42d^+^GFP^+^）占比；B：移植后受体小鼠外周血中供体来源血小板计数（*n*＝9）；C：移植后受体小鼠外周血中供体来源血小板占比（*n*＝9）

3. 供体巨核细胞来源血小板的形态：为了从形态特征角度对供体巨核细胞产生的血小板进行评估，我们对比了移植后96 h供体来源的GFP^+^血小板与受体自身产生的GFP^−^血小板的形态及大小。首先，免疫荧光观察发现，外周血GFP^+^血小板与GFP^−^血小板均呈现规则圆形或长圆形，在形态上均无明显差异（图5A）。进一步直径统计显示，GFP^+^血小板的直径及其分布范围与受体自身生成的GFP^−^血小板均无统计学差异（图5B）。流式细胞术分析也证明，两组血小板的大小分布无明显差异（图5C）。上述结果表明，供体来源巨核细胞所产生的血小板具有正常的形态和大小。

**图5:**
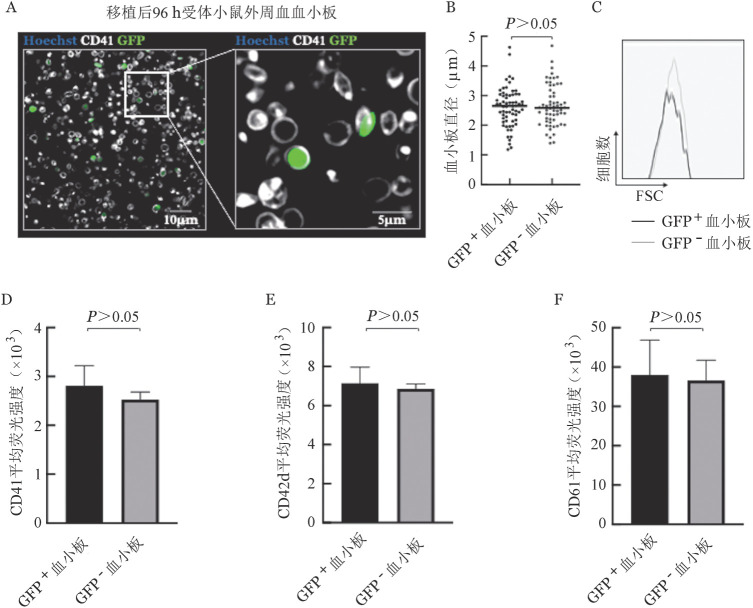
移植后受体小鼠外周血中供体来源血小板形态与表面标志分子表达水平 GFP：绿色荧光蛋白。A：移植后96 h小鼠外周血血小板免疫荧光染色（Hoechst标记细胞核，CD41为血小板表面标志分子，GFP为供体小鼠细胞自发荧光）；B：移植后96 h受体小鼠外周血血小板直径分布；C：移植后96 h受体小鼠外周血血小板前向角散射（FSC）对比；D、E、F：流式细胞术检测移植后96 h供体来源血小板表面标志分子表达（*n*＝4）

4. 流式细胞术检测供体巨核细胞来源血小板表面标志物表达：CD41、CD42d和CD61是血细胞表面经典的标志分子，为了从分子特征角度对供体巨核细胞来源血小板进行评估，我们在移植后96 h对受体小鼠血小板表面标志分子的表达情况进行检测。结果显示，CD41、CD42d和CD61在供体来源血小板与受体来源血小板中的平均荧光强度均无明显差异（图5D、E、F），表明供体巨核细胞产生的血小板具有正常的标志分子表达水平。

## 讨论

相较于既往巨核细胞产生血小板模型，本研究在诸多方面进行了创新与优化。首先，在供体细胞的选择上，采用了较温和的磁珠筛选方式，富集小鼠骨髓原代巨核细胞作为供体细胞。在以往研究中，由于巨核细胞体内数量稀少、胞体较大、胞质易破碎等原因，多利用诱导分化体系获得体外培养的巨核细胞构建血小板产生模型。早期，该类体外巨核细胞培养体系的主要细胞来源有成人或脐血来源CD34^+^细胞、人胚胎干细胞、诱导多能干细胞[6]–[8],[10]–[12]等。但由于血小板产生效率低、细胞成本高昂、培养周期长以及残留的胚胎期属性难以反映成年巨核细胞功能[10]–[11],[13]等限制，体外培养难以真实、客观地反映体内血小板产生过程。因此，本模型以原代巨核细胞直接作为血小板产生起点，不仅能够避免过多人为因素对巨核细胞血小板产生过程造成干扰，同时还可以缩短研究周期，在移植后72 h开始便可以观察到供体来源血小板的产生，相较于体外诱导干祖细胞向巨核谱系分化至血小板产生所需要的15～20 d[6]更为高效、便捷。

其次，在移植方式上，本研究通过骨髓腔注射直接将巨核细胞移植入成年小鼠最主要的血小板产生部位——骨髓，最大程度还原了骨髓原位血小板产生的过程。现阶段体内模型构建手段主要为尾静脉输注，但经尾静脉输注的大部分巨核细胞会聚集于肺脏并产生血小板，并未到达骨髓而重现正常生理状态下的骨髓腔血小板产生过程[14]。本研究利用骨髓腔注射进行巨核细胞移植，既能避免巨核细胞在循环中的损失，又可以规避肺脏等“异位血小板产生”对骨髓原位血小板产生的干扰，因此能够更为真实地还原巨核细胞在骨髓产生血小板的过程。

近年来，随着单细胞测序技术的发展，对于巨核细胞异质性的解析逐渐深入。依据其转录组特征，不同的巨核细胞亚群可能存在血小板产生潜能的差异[15]–[18]，但对各巨核细胞亚群血小板产生差异的探究至今仅停留在转录组层面，缺乏有力的功能手段进行验证。而本研究引入磁珠对巨核细胞进行富集可以为之后探索不同亚群之间的差异奠定基础。例如通过叠加不同的标志分子磁珠对不同亚群巨核细胞进行更为精准的分离富集，进而对比不同亚群血小板产生的效率、方式、子代血小板功能及特性等，以此为巨核细胞亚群的鉴定以及后续功能验证予以支持。

另一方面，除了在生理条件下对血小板产生过程进行研究，该模型还可以与各疾病小鼠模型相结合，用于探索不同疾病状态下巨核细胞产生血小板的差异。例如通过选择不同的供受体小鼠，既可以将各疾病状态下巨核细胞移植入正常受体小鼠以评估巨核细胞本身及其子代血小板的功能，也可以将正常巨核细胞移植入疾病模型等不同受体小鼠体内来探究造血微环境对巨核谱系造血的影响。此外，近年来，巨核细胞在机体免疫防御、多发性骨髓瘤等良恶性肿瘤疾病中均被证实发挥重要作用[19]–[24]。利用本研究所构建的骨髓腔移植模型，不仅能够单纯通过子代血小板的检测来评估巨核细胞在血小板产生过程中的差异，结合各类炎症指标、肿瘤标志物等检测，同样可以反映巨核细胞在不同病理状态下所发挥的除血小板产生之外的功能，从而为巨核细胞功能多样性的验证提供工具。

综上，本研究所构建的原代巨核细胞骨髓腔血小板产生模型可以为评估巨核细胞血小板产生能力及探究血小板产生调控机制提供有力的工具，但目前仍存在巨核细胞富集纯度低、祖细胞混杂等问题，后续需要进一步通过更为先进的分选或叠加更为特异的磁珠组合等手段优化巨核细胞纯化方式，以满足更为细致精准的巨核细胞功能验证需求，最终实现巨核细胞异质性的解析以及其在分子层面与功能层面的对接。
